# Long-term sevoflurane exposure relieves stress-enhanced fear learning and anxiety in PTSD mice

**DOI:** 10.1515/tnsci-2022-0313

**Published:** 2023-10-28

**Authors:** Ying Du, Minhui Xu, Yan Su, Yujia Liu, Yiming Zhou, Xiaoping Gu, Tianjiao Xia

**Affiliations:** Department of Anesthesiology, Affiliated Drum Tower Hospital, Medical School of Nanjing University, Nanjing, China; Medical School, Nanjing University, Nanjing, China; Jiangsu Key Laboratory of Molecular Medicine, Nanjing University, Nanjing, China; State Key Laboratory of Pharmaceutical Biotechnology, Nanjing University, Nanjing, China

**Keywords:** PTSD, sevoflurane, stress, memory impairment

## Abstract

**Objectives:**

Post-traumatic stress disorder (PTSD) is characterized by recurrent episodes of severe anxiety after exposure to traumatic events. It is believed that these episodes are triggered at least in part by environmental stimuli associated with the precipitating trauma through classical conditioning, termed conditioned fear. However, traditional methods of conditioned fear memory extinction are frequently ineffective for PTSD treatment due to the contribution of non-associative sensitization caused by trauma. Anesthetics have shown promise for treating various psychiatric diseases such as depression.

**Methods:**

In this study, we examined if the inhaled anesthetic sevoflurane can suppress stress-enhanced fear learning (SEFL) in PTSD model mice. Model mice exposed to 2.4% sevoflurane for 6 h exhibited reduced freezing time and behavioral anxiety compared to sham-treated model mice. To explore the underlying mechanisms, we evaluated the regional expression levels of glucocorticoid receptors (GRs), cannabinoid CB1 receptors (CB1Rs), D1 dopamine receptors (D1Rs), and D2 dopamine receptors (D2Rs).

**Results:**

We verified that both GR and CB1R were significantly upregulated in the hippocampus, amygdaloid nucleus, and prefrontal cortex (PFC) of model mice, while D1R and D2R were downregulated. All of these expression changes were partially normalized in the PFC by 6 h but not with 2 h sevoflurane exposure.

**Conclusions:**

These results showed that sevoflurane exposure following traumatic events may be an effective treatment for PTSD.

## 1 Introduction

Post-traumatic stress disorder (PTSD) is a chronic and debilitating psychiatric illness induced by the experience of life-threatening events or catastrophic psychological trauma [1]. It is characterized by recurrent episodes of severe fear and anxiety that interfere with daily life and are frequently difficult to treat [2]. Both associative learning from the pairing of stimuli during the precipitating event and non-associative sensitization are implicated in PTSD pathogenesis [3]. Extinction-based therapies can ameliorate the associative fear [4] but are often of limited efficacy for reducing non-associative symptoms produced by trauma [5]. Thus, non-associative fear memories could be a significant source of refractory symptoms, necessitating the development of alternative treatments.

Anesthetics and sedatives have shown to be promising for the treatment of other neurological and psychiatric disorders, suggesting potential utility for the non-associative symptoms of PTSD. Sevoflurane is a widely applied inhaled anesthetic that induces amnesia, unconsciousness, and immobility [6], and several inhaled anesthetics including sevoflurane have shown cytoprotective efficacy at low doses [7,8]. For instance, it has been reported that sevoflurane can protect against ischemia/reperfusion injury and immune-related tissue injury by enhancing antioxidant capacity and immune modulation [9,10,11]. However, there have been a few studies examining the potential application of sevoflurane for psychiatric diseases. In our previous study, we reported that sevoflurane exposure for several hours promoted the extinction of fear memory in mice exposed to repeated pairing of sound (the conditioned stimulus) with shock (an unconditioned stimulus) [12], suggesting potential therapeutic value for PTSD. Given that extinction training is ineffective in many PTSD patients, we hypothesized that sevoflurane may also diminish non-associative sensitization. Therefore, in the current study, we established a stress-enhanced fear learning (SEFL) model of PTSD and re-evaluated the effects of sevoflurane administration.

## 2 Materials and methods

### 2.1 Animals

Male C57BL/6 mice (8–10 weeks and weighing 20–25 g) were used in the experiment. All mice were housed with a controlled 12-h light/dark cycle (lights turned on at 8:00) and were accessible to food and water for at least 2 weeks before any interference was performed. The room temperature was kept at 20–22°C, and the room humidity was maintained at 50–60%.

In this study, the mice were sacrificed by using euthanasia boxes (Yuyan instruments, LC500). First, the instructions “Connection Diagram” were followed to connect the pipeline. Then, the output pressure was adjusted to 0.2–0.5 MPa. The mice were then placed in the euthanasia box and locked in the snap. Finally, the CO_2_ gas flow meter was adjusted. Through flow control, the concentration of CO_2_ in the chamber was gradually increased, reducing the time the mice felt pain and suffering.

### 2.2 PTSD model

The PTSD model was derived from Rau’s previous study with minor modifications [3]. This experiment was conducted using a fear conditioning experimental system (Panlab, Spain). On the first day, mice were placed in a new context (A) and then were given six foot-shocks (0.8 mV), which simulated a stressful or “traumatic” event. The next day, we put the mice in a different context (B), and they were given a single foot-shock, which referred to a reminder of the original stressful event. On day 3, mice were returned to context B for 512 s to test their fear to context B in the absence of shock. However, the Ctrl group in this model received no electric shocks on day 1 during the context A procedure and single foot-shock on day 2 during the context B procedure. The fear memory was estimated as freezing time and the PTSD mice should increase significantly in freezing time compared with the control group. Context A and context B were distinguished by different coarseness, flavor, and brightness. During context A, a single high-frequency sound (4,000 Hz, 80 dB) was produced while white noise (80 dB) was supplied in context B. At the end of the experiment, the mice were returned to the cage. The test box was wiped with 75% alcohol at the end of each test to avoid the impact of other mouse odors on the experiment.

### 2.3 Anesthesia

The anesthesia box air inlet was connected to the anesthesia machine (Remain, China) gas evaporation tank, consisting of an air connection with an anesthesia monitor (Drager, Germany) for monitoring the sevoflurane concentrations (Jiangsu Xinchen Pharmaceutical Co., Ltd., China).

A 1.0 MAC (minimal alveolar concentration) sevoflurane anesthesia was chosen in our study. Animal anesthesia was performed using an inhalant gas mixture of sevoflurane and air in reference to our previous study. Mice were placed in a chamber with 2.4% sevoflurane (Jiangsu Xinchen Pharmaceutical Co., Ltd., China) in 100% oxygen at a flow rate of 2 L/min for maintenance for 6 h (from 21:00  to 3:00 in the dark cycle). During sevoflurane exposure, an anesthesia monitor was used to continuously monitor the concentration of isoflurane in the chamber, and respiration was observed to prevent respiratory depression. Mice were placed on a heating pad support to keep the body temperature within 36.5 ± 0.5°C, monitored with a rectal temperature probe. The same procedure was performed for the control animals but not exposed to the isoflurane. Mice in the control group were placed into the container and were exposed to air only.

**Figure 1:**
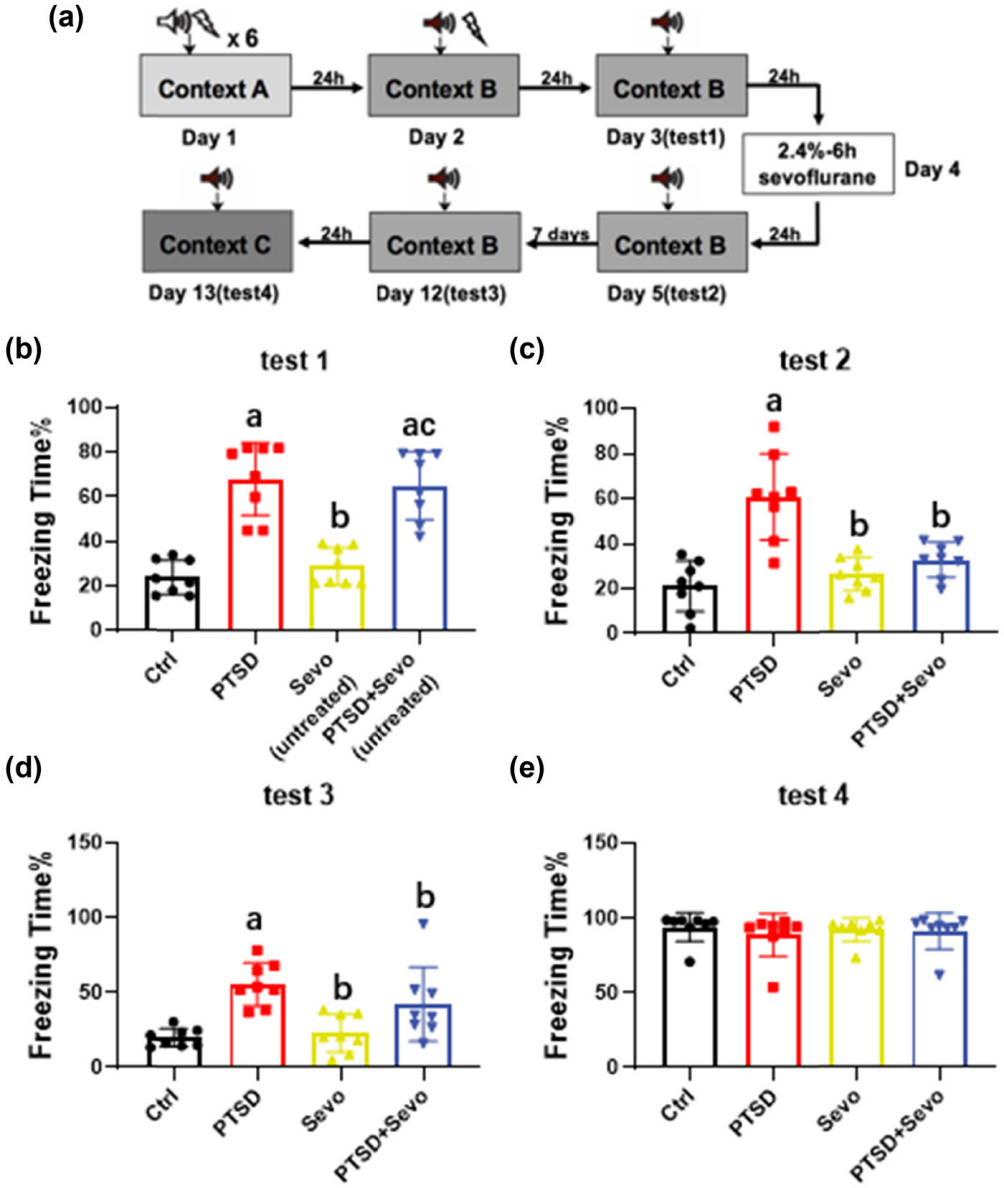
Effects of long-term sevoflurane exposure on the fear performance of PTSD mice. (a) The flow chart. (b) The freezing time in test 1 after PTSD modeling. (c) The freezing time in test 2 after 6 h of sevoflurane inhaling. (d) The freezing time in test 3 after 7 days of test 2. (e) The activity time in test 4 of memory generalization. Data are expressed as mean ± SD, *N* = 8. a *p* < 0.05 vs control group, b *p* < 0.05 vs PTSD group, c *p* < 0.05 vs sevo (untreated) group. No excluded mice.

We utilized the same mice for both the behavioral and neurochemical analyses. The animals were killed right after test 4 (Figure 1) with an overdose of isoflurane. Then, the fresh brains were dissected without perfusion for WB. Separate cohorts were used for 6 and 2 h PTSD experiments, whereas, for the open-field and forced swim test, we used a new cohort for both tests.

### 2.4 Open-field test

The open-field test was conducted in an uncovered plastic chamber (50 cm × 50 cm × 40 cm). All the animals were placed in the center of the floor to make sure they had free access to explore the chamber. Each mouse explored freely in 10 min. The activity time of the center area and the periphery area were, respectively, recorded by an automated video-tracking system (Biobserve, Bonn, Germany). The percentage of time in the center was defined as the percent time spent in the central 30 cm × 30 cm area of the open field. The total distance traveled in 10 min was calculated (in cm).

### 2.5 Forced swimming test

The forced swimming test was conducted in a cylindrical container containing water (23 ± 1°C). The diameter and height of the container were 10 cm and 25 cm. The water was up to 19 cm bottom to make mice swim freely in it. All the mice had to swim for 6 min and only the last 4 min were defined as the test stage to avoid unstable immobility behavior in the first 2 min.

Mice were considered freezing when they stopped swimming and only kept their heads above the water and appeared to float on water. The percentage of freezing time was calculated and analyzed among groups.

### 2.6 Western blot

The samples were homogenized in radioimmunoprecipitation assay lysis buffer (Beyotime, Shanghai, China) with a mixture of protease and phosphatase inhibitor cocktail (Abcam, Cambridge, UK). Proteins (20 μg) were separated on a sodium dodecyl sulfate-polyacrylamide gel electrophoresis gel and transferred to polyvinylidene difluoride membrane filters (Millipore, Burlington, MA). The blots were blocked with 5% bovine serum albumin and incubated with primary antibodies overnight at 4°C. The following primary antibodies were used: anti-β-actin (1:1,000 dilution; Abcam), anti-GADPH (1:1,000 dilution; Abcam), anti-cannabinoid CB1 receptor (anti-CB1R; 1:1,000 dilution; Abcam), anti-D1 dopamine receptor (anti-D1R; 1:1,000 dilution; Abcam), anti-D2 dopamine receptor (anti-D2R; 1:1,000 dilution; Abcam), and anti-glucocorticoid receptor (anti-GR; 1:1,000 dilution; Abcam). The membranes were washed in Tris-buffered saline with Tween 20 buffer and incubated with anti-mouse horseradish peroxidase (HRP)-conjugated secondary antibody (1:10,000 dilution; Abcam) or anti-rabbit HRP-conjugated secondary antibody (1:10,000 dilution; Abcam) for 2 h at room temperature. Protein bands were visualized with a Western Chemiluminescence HRP Substrate kit (Millipore) using a Tanon 5200 (Tanon Science & Technology, Shanghai, China). Relative intensities of the specific protein bands normalized to β-actin or GADPH were quantified using ImageJ software (National Institute of Health, Bethesda, MD, USA).

### 2.7 Statistical analysis

All data are presented as mean ± SD. Statistical analyses were performed using a computerized statistical package (SPSS 22.0) and GraphPad Prism software version 7.0 (GraphPad Software, Inc., San Diego, CA, USA). One-way ANOVA followed by Bonferroni post hoc tests was used to evaluate the quantitative differences and the behavioral data. Differences were deemed statistically significant at probability values (*p*) of <0.05.


**Ethical approval:** The research related to animals’ use complied with all the relevant national regulations and institutional policies for the care and use of animals. This study was approved by the Nanjing University Institutional Animal Care and Use Committee, in accordance with international regulations. The study is reported in accordance with ARRIVE guidelines. All processing methods were in line with Directive 2010/63/EU.

## 3 Results

### 3.1 Long-term sevoflurane exposure suppressed SEFL in PTSD mice

In test 1 on day 3, PTSD mice showed significantly enhanced freezing time (68.12 ± 16.2%, *p* < 0.001 vs Ctrl and Sevo [untreated], Figure 1a and b), which indicated successful modeling of mice shocked six times seriously in context A on day 1. After being exposed to sevoflurane for 6 h, mice in the PTSD + Sevo group exhibited decreased freezing time compared with PTSD (32.59 ± 8.35% vs 60.74 ± 20.84, *p* < 0.001, Figure 1a and c) in test 2, which was designed for evaluating therapeutic effects of sevoflurane on PTSD mice, and our results show a positive response to it. To further explore the effective time of sevoflurane, we repeated test 3 after 7 days and the results were the same as before (28.03 ± 10.08% vs 55.62 ± 14.33, *p* < 0.01 vs PTSD, Figure 1a and d). In addition, we performed test 4 in order to exclude the influence of memory generalization in this PTSD model. It can be observed that all groups showed a high degree of activity without fear in context C (*p* > 0.05, Figure 1a and e).

### 3.2 Long-term sevoflurane exposure ameliorated anxiety in PTSD mice

**Figure 2:**
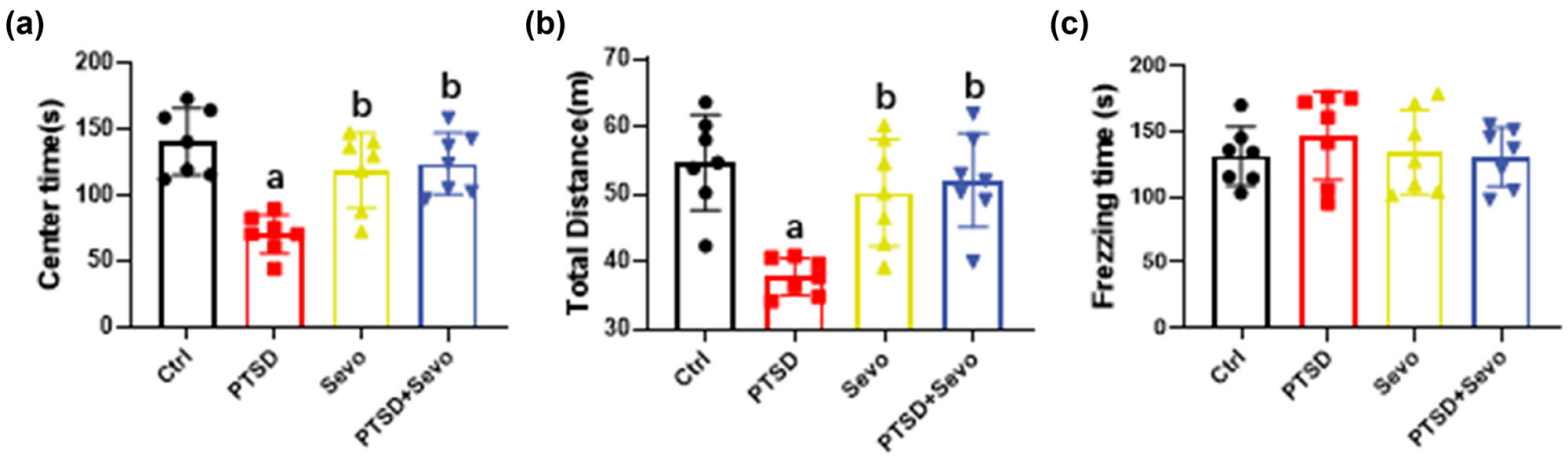
Effects of long-term sevoflurane exposure on the anxiety of PTSD mice. (a) Time spent by mice in the center space in the open-field test. (b) Total distance run by the mice in the open-field test. (c) The freezing time of mice in the forced swimming test. Data are expressed as mean ± SD, *N* = 8. a, *p* < 0.05 vs control group, b, *p* < 0.05 vs PTSD group. No excluded mice.

To further investigate the effects of sevoflurane on anxiety in PTSD mice, we conducted the open-field test on day 12 (7 days after anesthesia). PTSD mice showed reduced time spent in the center of the arena compared with other groups (70.58 ± 16.28, *p* < 0.001 vs Ctrl, Sevo, and PTSD + Sevo, Figure 2a). Similar data could be seen in the total distance traveled by PTSD mice (37.72 ± 2.98, *p* < 0.001 vs Ctrl, Sevo, and PTSD + Sevo, Figure 2b). In the forced swimming test, the freezing time was similar among all groups (*p* > 0.05, Figure 2c).

### 3.3 Short-term sevoflurane exposure improved SEFL transiently in PTSD mice

**Figure 3:**
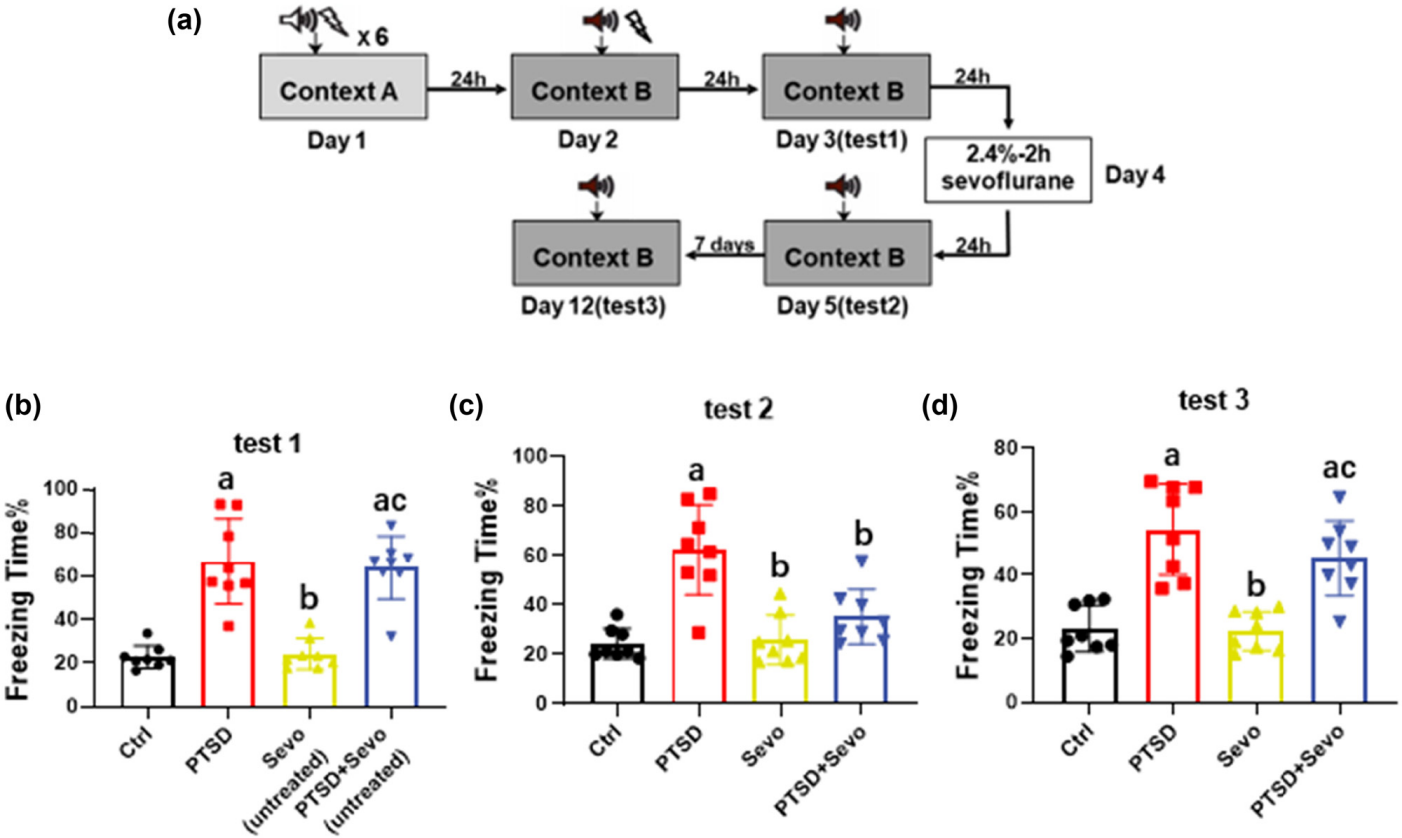
Effects of short-term sevoflurane exposure on the fear performance of PTSD mice. (a) The flow chart. (b) The freezing time in test 1 after PTSD modeling. (c) The freezing time in test 2 after 2 h of sevoflurane inhaling. (d) The freezing time in test 3 after 7 days of test 2. Data are expressed as mean ± SD, *N* = 8. a *p* < 0.05 vs control group, b *p* < 0.05 vs PTSD group, c *p* < 0.05 vs sevoflurane group. No excluded mice.

For further investigating whether short-term sevoflurane inhaled could induce SELF either, we repeated experiment 1, except that the duration of anesthesia was shortened to 2 h. In test 1, the data showed obviously an enhanced freezing time in the PTSD group (66.93 ± 19.78%, *p* < 0.001 vs Ctrl and Sevo [untreated], Figure 3a and b). After being exposed to sevoflurane for 2 h, mice in the PTSD + Sevo group exhibited decreased freezing time compared with PTSD (35.24 ± 11.12% vs 62.41 ± 18.28, *p* < 0.001, Figure 3a and c) in test 2. We repeated test 2 after 7 days and the results were modified. The PTSD mice still showed higher freezing times than the control group (54.50 ± 14.34% vs 23.21 ± 7.30, *p* < 0.01, Figure 3a and d), while mice exposure to sevoflurane after 7 days could not revise the effect (45.34 ± 11.75%, *p* > 0.05 vs PTSD, *p* < 0.05 vs Ctrl and Sevo, Figure 3a and d).

### 3.4 GR and CB1R expressions were increased in brain areas in PTSD mice and sevoflurane normalized this effect

**Figure 4:**
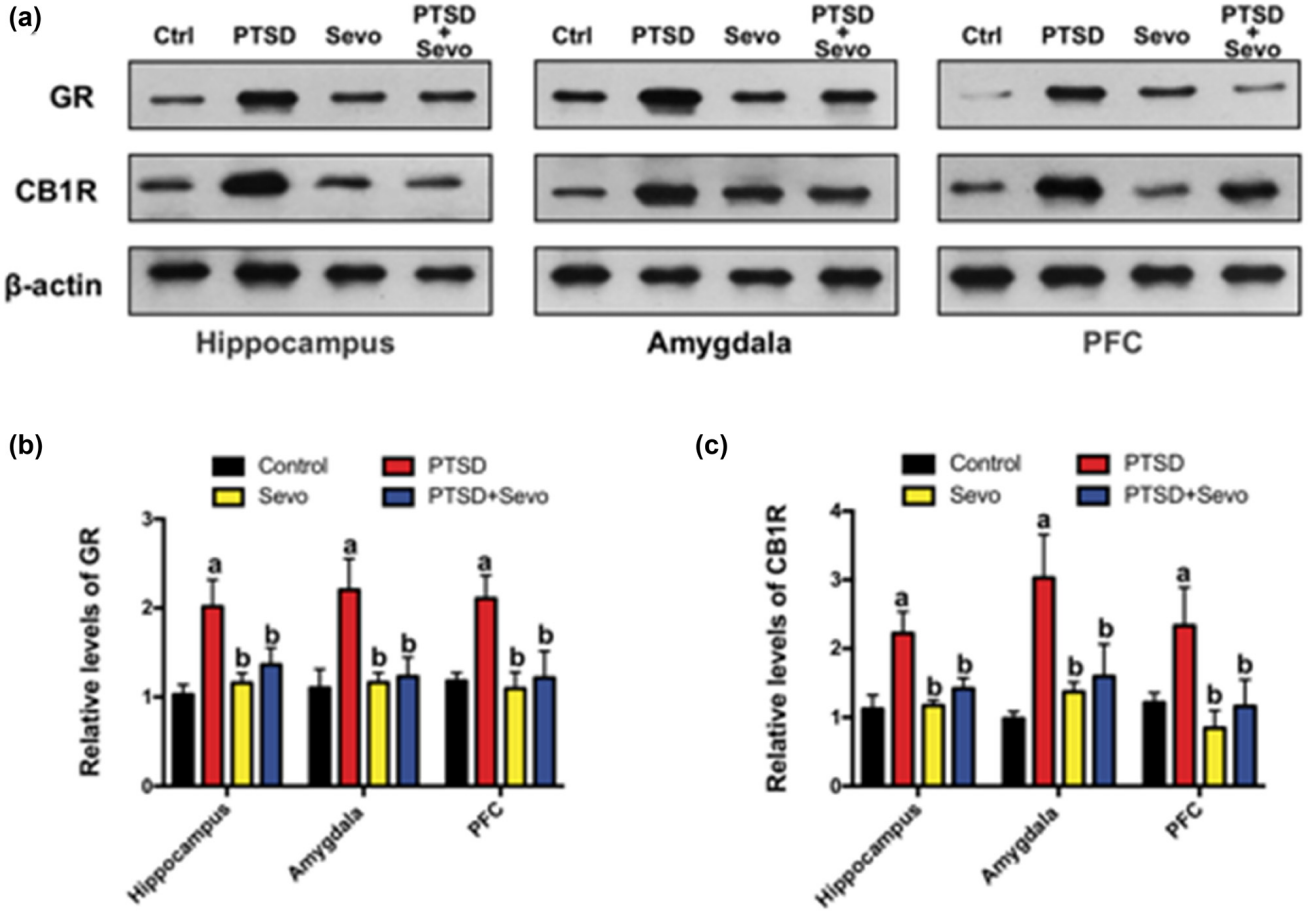
Changes in GR and CB1R expression in the hippocampus, amygdala, and PFC. (a) Representative blots of each protein. (b and c) Statistical analysis of the relative protein expression. Data are expressed as mean ± SD, *N* = 3. a *p* < 0.05 vs control group, b *p* < 0.05 vs PTSD group.

We explored the levels of relative GR and CB1R expressions in the hippocampus, amygdala, and prefrontal cortex (PFC) in all groups. In the PTSD group, the expression of GR was upregulated in the hippocampus (2.02 ± 0.30), amygdala (2.20 ± 0.35), and PFC (2.11 ± 0.26) compared with the control and Sevo groups (*p* < 0.01 vs Ctrl and Sevo). Sevoflurane administered for 6 h modified these alterations in the three brain areas (hippocampus: 1.36 ± 0.19, amygdala: 1.23 ± 0.22, PFC: 1.22 ± 0.30, *p* < 0.05 vs PTSD, Figure 4a and b). Similar changes were seen in the CB1R relative expression. Higher expression of CB1R was found in the PTSD group in the hippocampus (2.22 ± 0.22), amygdala (3.03 ± 0.63), and PFC (2.33 ± 0.56) compared with the control and Sevo groups (*p* < 0.01 vs Ctrl and Sevo). About 6 h of sevoflurane treatment normalized the alterations in the three brain areas (hippocampus: 1.41 ± 0.15, amygdala: 1.60 ± 0.47, PFC: 1.16 ± 0.39, *p* < 0.05 vs PTSD, Figure 4a and c).

### 3.5 Expression of D1/2 dopamine receptors decreased after modeling and sevoflurane normalized this change partly in the PFC

**Figure 5:**
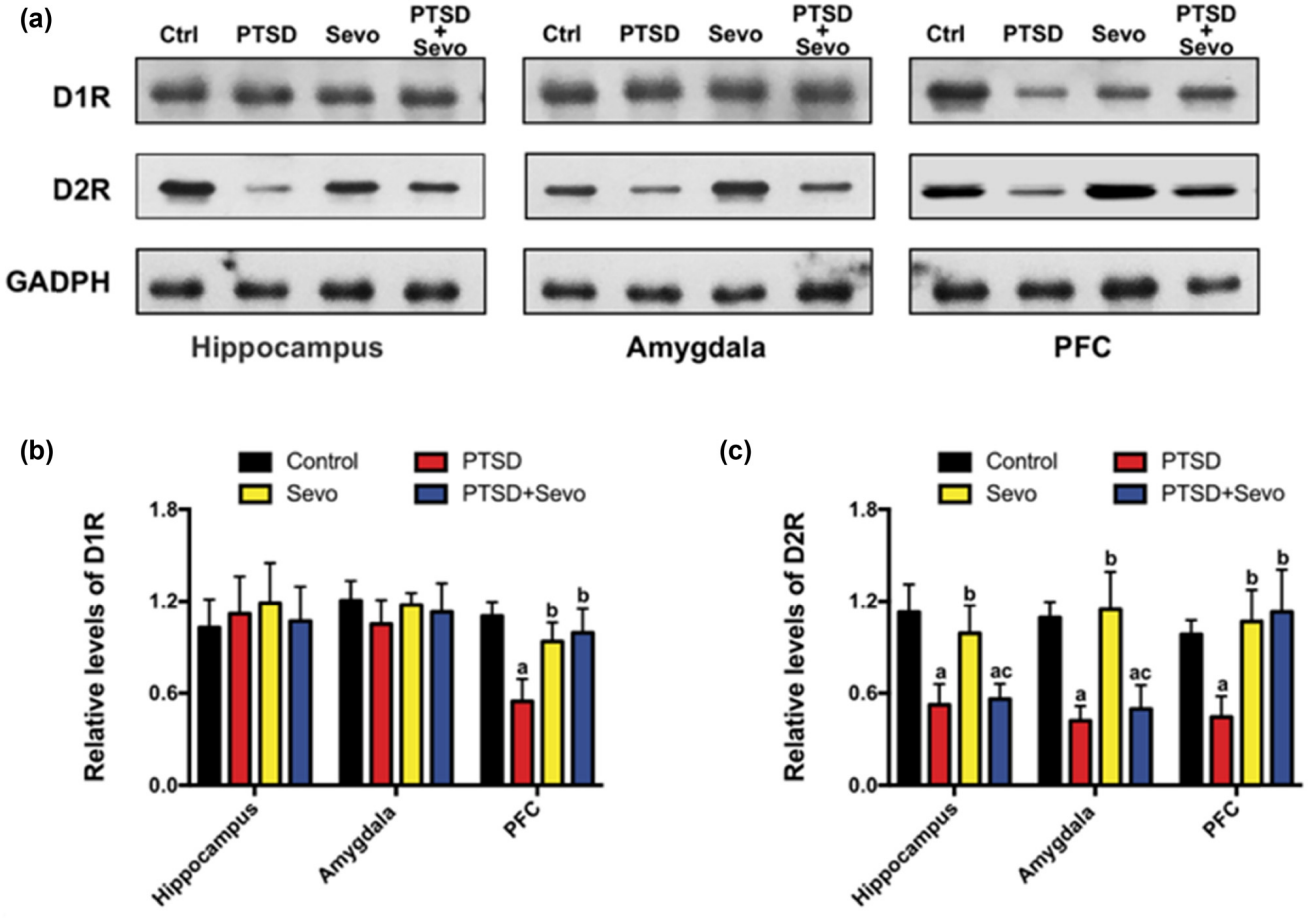
Changes in D1R and D2R expression in the hippocampus, amygdala, and PFC. (a) Representative blots of each protein. (b and c) Statistical analysis of the relative protein expression. Data are expressed as mean ± SD, *N* = 3. a *p* < 0.05 vs control group, b *p* < 0.05 vs PTSD group.

We next investigated the expression of D1R and D2R in the hippocampus, amygdala, and PFC in all groups. No alterations in D1R were observed in the hippocampus (1.11 ± 0.25) or amygdala (1.05 ± 0.16) of PTSD mice (*p* > 0.05 vs all groups, Figure 5a and b). In the PFC, a significant group effect was found for D1R in the PTSD group (0.55 ± 0.15, *p* < 0.05 vs Ctrl, Sevo) and the sevoflurane inhalation increased the level (0.94 ± 0.13, *p* < 0.05 vs PTSD, Figure 5a and b). However, in the case of D2R, it changed obviously. The level of D2R was decreased in the hippocampus (0.52 ± 0.14), amygdala (0.42 ± 0.10), and PFC (0.44 ± 0.14) in the PTSD mice (*p* < 0.01 vs Ctrl and Sevo). Sevoflurane did not reverse the downregulation very well. Only in the PFC, sevoflurane could improve the level of D2R (1.13 ± 0.27, *p* < 0.05 vs PTSD, Figure 5a and c). No differences were observed in the hippocampus (0.56 ± 0.10) and amygdala (0.50 ± 0.15) compared with the PTSD group (*p* > 0.05, Figure 5a and c).

## 4 Discussion

The SEFL animal model of PTSD demonstrated robust fear in context B on day 3 post-induction, confirming that shock exposure (trauma) can induce a non-associative fear response. Notably, 2.4% sevoflurane administration for 6 h on day 4 after modeling significantly attenuated SEFL and anxiety for at least 7 days, while 2 h of exposure had only a transient suppressive effect. This study suggests that a single exposure to sevoflurane several days after trauma can partially ameliorate PTSD symptoms, presumably by normalizing the changes in the transmitter signaling triggered by physiological responses and memories of the traumatic event.

The anesthetic ketamine was recently approved by the United States Food and Drug Administration (FDA) for the treatment of major depression, and there is growing interest in the use of other anesthetics in clinical psychology and psychiatry, especially for memory-related diseases such as PTSD [13,14]. Sevoflurane is generally considered to have milder effects on cognitive function than isoflurane [15,16,17], suggesting greater safety and tolerability as a potential treatment for PTSD. Indeed, the SEFL model examined in this study demonstrated persistent PTSD-like symptoms that were suppressed by a sufficient sevoflurane dose administered several days after the traumatic event (exposure to repeated electrical shocks). A previous study reported that sevoflurane inhalation during trauma reduced subsequent SEFL in mice [18], but such preventative use is impractical as treatment, so our demonstration that sevoflurane can be used to reverse the deleterious effects of exacerbated non-associative fear memory has potential clinical significance. These findings showed that sevoflurane exposure following traumatic events may be an effective treatment for PTSD. This salutary effect was dose-dependent, as a 2 h administration significantly improved SEFL only on the first day post-treatment while the treatment effect of the 6 h administration lasted at least 7 days. This sustained effect is also critical because repeated administration of inhaled anesthetics can induce brain damage in mice and humans [19,20]. However, we only tested two dose regimens (2 and 6 h at a minimum alveolar concentration of 1.0), so other dose regimens should be tested to identify the most suitable starting parameters for subsequent studies.

Exposure to stress upregulates the expression of GR in brain regions implicated in fear memory such as the hippocampus, amygdala, and PFC, while administration of GR antagonists can prevent the neuronal damage caused by stress [21,22]. In accordance with previous studies, GR expression was elevated in PTSD model mice in the hippocampus, amygdala, and PFC, while inhalation of sevoflurane reversed this upregulation but had no effect on basal GR expression in control mice. In addition to dysregulation of GR expression, PTSD is reported to alter the expression of CB1R, a receptor widely expressed in both limbic structures and hypothalamic nuclei where it can modulate GR signaling [23,24]. In the current study, the traumatic experience upregulated CB1R expression, and this response was reversed by sevoflurane. In contrast, a previous study reported that CB1R was downregulated during protracted contextual fear memory and that a CB1R agonist suppressed fear memory [25,26]. This discrepancy may result from the different modeling parameters used in these studies, such as the stress-induction stimulus, intensity, and repetition. Overall, these results showed that sevoflurane exposure following traumatic events may regulate the expression level of the GR and CB1R to alleviate SEFL. For instance, the six shocks in context A actually reduced durable sensitization among PTSD model mice in the aforementioned study.

In addition to effects on glucocorticoid signaling, CB1R has also been reported to modulate the expression of dopamine receptors, which in turn can regulate fear memory [27]. Genes related to D2 receptor signaling have also been linked to an increased risk of PTSD [28]. Further, the amygdala–hippocampal–cortical pathway is responsible for processing and storing fear-related memories and for coordinating fear-related behaviors [29]. Thus, we measured the levels of D1R and D2R in hippocampus, amygdala, and PFC. While D1R expression was relatively stable, D2R expression was substantially reduced in model mice. Sevoflurane reversed this effect in the PFC but not in the hippocampus and amygdala, possibly because the PFC is involved in the storage of long-term fear memory [30] and expression was not examined until 13 days after the trauma. Moreover, the exacerbated reaction to shock-related cues may last for months, suggesting marked changes in the PFC [31]. Additional studies are required to fully elucidate the spatiotemporal changes in neural D2R, GR, and CB1R expression levels following trauma and various post-traumatic treatments.

## 5 Conclusion

The present study provides support for the effectiveness of sevoflurane as a suppressor of SEFL and anxiety in PTSD model mice. This sevoflurane treatment model may also help unravel the neurocellular and molecular mechanisms underlying PTSD.
